# The genomic landscape of ANCA-associated vasculitis: Distinct transcriptional signatures, molecular endotypes and comparison with systemic lupus erythematosus

**DOI:** 10.3389/fimmu.2023.1072598

**Published:** 2023-03-27

**Authors:** Aggelos Banos, Konstantinos Thomas, Panagiotis Garantziotis, Anastasia Filia, Nikolaos Malissovas, Antigone Pieta, Dimitrios Nikolakis, Alexandros G. Panagiotopoulos, Aglaia Chalkia, Dimitrios Petras, George Bertsias, Dimitrios T. Boumpas, Dimitrios Vassilopoulos

**Affiliations:** ^1^ Laboratory of Autoimmunity and Inflammation, Center for Clinical, Experimental Surgery and Translational Research, Biomedical Research Foundation of the Academy of Athens, Athens, Greece; ^2^ Clinical Immunology- Rheumatology Unit, 2nd Department of Medicine and Laboratory, General Hospital of Athens Ippokrateio, School of Medicine, National and Kapodistrian University of Athens, Athens, Greece; ^3^ Department Rheumatology and Immunology, Hannover Medical School, Hannover, Germany; ^4^ Rheumatology and Clinical Immunology Unit, 4th Department of Internal Medicine, Attikon University Hospital, Athens, Greece; ^5^ Amsterdam Institute for Gastroenterology Endocrinology and Metabolism, Department of Gastroenterology, Academic Medical Center, Amsterdam University Medical Centers (UMC), University of Amsterdam, Amsterdam, Netherlands; ^6^ Department of Rheumatology and Clinical Immunology, Amsterdam Rheumatology & Immunology Center (ARC), Amsterdam University Medical Centers (UMC), University of Amsterdam, Amsterdam, Netherlands; ^7^ Amsterdam Institute for Infection & Immunity, Department of Experimental Immunology, Amsterdam University Medical Centers (UMC), University of Amsterdam, Amsterdam, Netherlands; ^8^ Nephrology Department, General Hospital of Athens Ippokrateio, Athens, Greece; ^9^ Department of Rheumatology and Clinical Immunology, University Hospital of Heraklion, Medical School, University of Crete, Heraklion, Greece; ^10^ Department of Immunity, Institute of Molecular Biology and Biotechnology-Foundation of Research and Technology-Hellas (FORTH), Heraklion, Greece; ^11^ Joint Academic Rheumatology Program, School of Medicine, National and Kapodistrian University of Athens, Athens, Greece

**Keywords:** autoimmune diseases, vasculitis, lupus (SLE), transcriptomics (RNA-seq), endotypes of disease

## Abstract

**Introduction:**

Anti-neutrophil cytoplasmic antibody (ANCA)-associated vasculitides (AAVs) present with a complex phenotype and are associated with high mortality and multi-organ involvement. We sought to define the transcriptional landscape and molecular endotypes of AAVs and compare it to systemic lupus erythematosus (SLE).

**Methods:**

We performed whole blood mRNA sequencing from 30 patients with AAV (granulomatosis with polyangiitis/GPA and microscopic polyangiitis/MPA) combined with functional enrichment and network analysis for aberrant pathways. Key genes and pathways were validated in an independent cohort of 18 AAV patients. Co-expression network and hierarchical clustering analysis, identified molecular endotypes. Multi-level transcriptional overlap analysis to SLE was based on our published data from 142 patients.

**Results:**

We report here that “Pan-vasculitis” signature contained 1,982 differentially expressed genes, enriched in leukocyte differentiation, cytokine signaling, type I and type II IFN signaling and aberrant B-T cell immunity. Active disease was characterized by signatures linked to cell cycle checkpoints and metabolism pathways, whereas ANCA-positive patients exhibited a humoral immunity transcriptional fingerprint. Differential expression analysis of GPA and MPA yielded an IFN-g pathway (in addition to a type I IFN) in the former and aberrant expression of genes related to autophagy and mRNA splicing in the latter. Unsupervised molecular taxonomy analysis revealed four endotypes with neutrophil degranulation, aberrant metabolism and B-cell responses as potential mechanistic drivers. Transcriptional perturbations and molecular heterogeneity were more pronounced in SLE. Molecular analysis and data-driven clustering of AAV uncovered distinct transcriptional pathways that could be exploited for targeted therapy.

**Discussion:**

We conclude that transcriptomic analysis of AAV reveals distinct endotypes and molecular pathways that could be targeted for therapy. The AAV transcriptome is more homogenous and less fragmented compared to the SLE which may account for its superior rates of response to therapy.

## Introduction

Anti-neutrophil cytoplasmic antibody (ANCA)-associated vasculitides (AAVs) include granulomatosis with polyangiitis (GPA), microscopic polyangiitis (MPA) and eosinophilic granulomatosis with polyangiitis (EGPA). They are associated with significant mortality (25% at five years after diagnosis) (1) and morbidity, due to disease- and treatment-related organ damage (2). AAV display a diverse clinical phenotype with multi-organ involvement including kidneys, upper and lower respiratory tract, nerves, joints, skin and the central nervous system. The organ involvement shows significant variation between patients and phenotypes, with 80% of renal-limited glomerulonephritis cases being positive for ANCA against myeloperoxidase (MPO+) and 90% of patients with ear, nose and throat involvement being positive for ANCA against proteinase-3 (PR3+) (3). Following induction therapy, more than 85% of patients will enter remission, however up to 40-50% of patients experience disease relapses despite maintenance immunosuppressive therapies (4). Given the complexity of the AAV natural course, the in-depth description of disease endotypes and the discovery of biomarkers to predict resistance to therapy, relapses or severe outcomes has become of paramount importance. To date, several candidate biomarkers have been tested for this purpose (i.e. B-cell count, ANCA type and titers) in randomized and observational trials, however most of them have come up with variable results (5–8). This better understanding of the underlying mechanisms of the disease has led to the introduction of novel therapies, such as those blocking the C5a receptor (avacopan) for AAV (9) and the anti-IFN I receptor (anifrolumab) for SLE.

AAV develop in genetically predisposed individuals following exposure to certain environmental factors (10). Genome-wide association studies (GWAS) have pointed to several major histocompatibility complex class II (MHC II) (11–13) and non-MHC (11, 12, 14) genes associated with AAV risk. Epigenetic mechanisms including DNA methylation, histone modification and therefore, expression regulation of key genes such as MPO and PRTN3 have also been described (15, 16). An interplay of several immune system components contributes to its pathogenesis, namely innate (neutrophil priming and activation, neutrophil extracellular traps (NETs) (17), alternative complement pathway) and adaptive immunity (CD4-induced B-cell stimulation and ANCA production by plasma cells, T helper 17 (Th17) cells forming and maintaining necrotizing granuloma, and finally quantitative and functional alteration of regulatory T cells (10, 18).

High throughput genomic technologies allow the systematic, comprehensive exploration of complex diseases without preconceived notions (19). Recently, using next-generation RNA sequencing we have defined signatures correlated with susceptibility, activity and severity in patients with systemic lupus erythematosus (SLE) and defined molecular endotypes of the disease (20). Different gene expression modules in SLE have been correlated with different clinical aspects. For example, CD4 and CD8 signatures were associated with disease outcome, whereas type 1 interferon response with disease activity (21). In AAVs, initial transcriptomic analyses revealed enrichment of genes implicated in IL7R pathway, TCR signaling and expansion of CD8 memory cells were associated with poor prognosis and higher relapse rates, whereas a distinct CD8 T-cell exhaustion signature correlated with low risk of relapse (21, 22). More recently, RNA sequencing approaches have offered additional insights suggesting a strong neutrophilic and lymphocyte signature (23, 24) some of them reminiscent of those in SLE. Yet, despite its aggressive course, in AAV responses to existing therapies are more solid than those in SLE. Comparison of the transcriptomic landscape of both diseases provide an unbiased look of the underlying pathogenetic mechanisms and explain the differences in the natural course and response to treatment.

Herein, we sought to define the transcriptomic signature of AAV patients, identify potential differences in RNA signatures between AAV subsets, define novel molecular endotypes, and compare its transcriptome to that of SLE.

## Materials and methods

### Patients

Thirty adult patients with AAV (GPA or MPA) followed in two tertiary referral hospitals (Hippokration General Hospital, HGH and Attikon University Hospital, AUH) were included. All patients fulfilled the Chapel Hill Consensus Conference definitions for GPA and MPA (25). Patients with EGPA were excluded due to its distinct pathogenesis and phenotype, in order to achieve a more homogenous cohort. The control group included 11 age- and sex-matched healthy individuals. All participants provided informed consent. The study was approved by the institutional review boards of both hospitals (HGH: 57/26-03-2018/AUH: 103/06-03-2014).

For each patient, the following data were collected: age at diagnosis and at sampling, sex, type of AAV (GPA/MPA) and ANCA status, Birmingham Vasculitis Activity Score for Wegener’s Granulomatosis (BVAS/WG) at diagnosis and at sampling, disease status at the time of sampling (active *vs*. remission), organ involvement, type of treatment at the time of blood sampling and, glucocorticoid dose (as prednisolone equivalent, mg/day). Active disease was defined as BVAS/WG >1. Disease remission was defined as BVAS/WG ≤ 1 for ≥6 months and daily prednisone dose of ≤10 mg (26). Relapse was defined as an at least 1-point increase in BVAS/WG in a patient previously in remission. Values are presented as mean ± standard deviation (SD) for continuous variables with normal distribution, median (interquartile range, IQR) for continuous nonparametric variables, and percentages for categorical variables (see **Table 1**).

**Table 1:** Demographics, clinical characteristics, and treatment at sampling of the 30 (discovery cohort) and 18 (validation cohort) AAV patients.

Variables	n (%)		
	Discovery cohort n = 30	Validation cohort n = 18	p
**Males, n (%)**	16 (53.3%)	8 (44.4%)	0.56
**Age at diagnosis, mean (SD)**	56.9 (14.9)	55.7 (17.7)	0.79
**Age at sampling, mean (SD)**	60.5 (14.1)	63.6 (17.2)	0.49
**AAV type, n (%)**			0.37
GPA	22 (73.3%)	11 (61.2%)	
MPA	8 (26.7%)	7 (38.8%)	
**ANCA status, n (%)**			0.49
cANCA/anti-PR3+	11 (36.7%)	9 (50.0%)	
pANCA/anti-MPO+	15 (50%)	8 (44.4%)	
Negative	4 (13.3%)	1 (5.6%)	
**BVAS/WG at diagnosis, median (IQR)**	6 (4-8)	5 (4-8.25)	0.76
**BVAS/WG at sampling, median (IQR)**	1 0 (4.25)	1 (0-2.75)	0.70
Organ involvement, n (%)			
Constitutional	18 (60%)	9 (50%)	0.49
Lung	27 (90%)	14 (77.8%)	0.24
Renal	21 (70%)	8 (44.4%)	0.08
ENT	15 (50%)	10 (55.5%)	0.71
Mucous/Eyes/Membranes	4 (13.3%)	3 (16.7%)	0.75
Nervous	5 (16.7%)	7 (38.9%)	0.08
Cutaneous	3 (10%)	3 (16.7%)	0.49
Carciovascular	1 (3.3%)	1 (5.6%)	0.99
**Disease status at sampling, n (%)**			0.32
Remission	13 (43.3%)	11 (61.1%)	
Active, newly diagnosed	8 (26.7%)	1 (5.6%)	
Active relapse	7 (23.3%)	5 (27.7%)	
Active, persistent	2 (6.7%)	1 (5.6%)	
Treatment type at sampling, n (%)			
No treatment	5 (16.7%)	5 (16.7%)	0.36
Corticosteroids	20 (66.7%)	10 (55.5%)	0.44
Cyclophosphamide	7 (23.3%)	0 (0%)	–
Rituximab	10 (33.3%)	8 (44.4%)	0.44
Azathioprine	9 (30%)	1 (5.5%)	0.09
Methotrexate	5 (16.7%)	0 (0%)	–
Mycophenolate mofetil	2 (6.7%)	2 (11.1%)	0.96
**Prednisone dose at sampling, median (IQR)**	3 (0-7.5)	3.75 (0-5)	0.85

Disease remission was defined as BVAS/WG ≤ 1 for ≥6 months and daily prednisone dose of ≤10 mg. Relapse was defined as an at least 1-point increase in BVAS/WG in a patient previously in remission. BVAS/WG, Birmingham Vasculitis Activity Score for Wegener’s Granulomatosis; SD, standard deviation (SD); IQR interquartile range, IQR.

### Isolation of total RNA

Whole blood samples were collected in PaxGene and Tempus RNA tubes. Total RNA was extracted using the Qiagen RNeasy kit and quantification was assessed using a NanoDrop spectometer. Quality control of RNA was assessed using the Agilent Bio Analyser.

### Library preparations and next‐generation sequencing

mRNA libraries were prepared using the Illumina TruSeq kit. 1x75bp single-end mRNA sequencing was performed on the Illumina NextSeq 500 in the BRFAA Greek Genome Center.

### Computational analysis of RNA sequencing data

Quality of sequencing was assessed using FastQC software (27). Raw reads in fastq format were aligned to the human reference genome (GRCh38.p12) by STAR mapper (28) and gene quantification was performed by HTSeq (29) using Gencode v29 annotation. Differential expression analysis was conducted using edgeR Bioconductor R package (quasi-likelihood linear model) (30). Statistically significant differentially expressed genes were considered those with a p-value of ≤0.05 and absolute fold change of >=1.5. Clustering of genes was performed using euclidean distance. Differential expressed genes for SLE analysis were extracted from published data as lists (20).

Enrichment analysis of DEGs and visualization were carried out using gProfiler (31), Enrichment Map (32), R Bioconductor packages, DOSE (33) and ReactomePA (34) and GeneMANIA (35) and GSEA (36). For all statistical comparisons, the cut-off for significance was set to 0.05 and p-values were adjusted for multiple comparisons.

Regulatory networks from the identified transcriptional signatures were constructed by applying the X2K Web algorithm (37), which creates a comprehensive network by integrating results from transcription factor enrichment analysis, protein-protein interaction network analysis, and kinase enrichment analysis (KEA) (38).

### Co-expression network analysis

Using the CoCena² (construction of co-expression network analysis-automated, https://github.com/UlasThomas/CoCena2), we identified modules of co-expressed transcripts. Disease molecular endotypes were determined using agglomerative hierarchical clustering of patients, based on their group fold changes (GFC) for each cluster of co-expressed genes. Enrichment analysis was performed using the clusterProfilerR package (39).

### qPCR validation

We performed qPCR for literature-curated genes as well as genes derived from gene ontology in an independent cohort of 18 AAV patients. Primer list is included as **Supplementary Table 1**.

## Results

### Patients

Thirty (30) AAV patients were included; 53.3% (16/30) were males, with a mean (± SD) age of 60.5 ± 14.1 years at the time of sampling. GPA was the most frequent clinical phenotype (22/30, 73.3%) whereas regarding ANCA status, 15 (50%) patients were pANCA/anti-MPO+, 11 (36.7%) were cANCA/anti-PR3+ and 4 (13.3%) were ANCA negative. Lung (90%), kidney (70%) and ENT (50%) were the more commonly affected organs. Thirteen (43%) patients were in remission. Patient, disease and treatment characteristics are presented in **Table 1**.

### Cytokine signaling and B-cell and T-cell abnormal function differentiate AAV patients from healthy individuals

**Figure 1:**
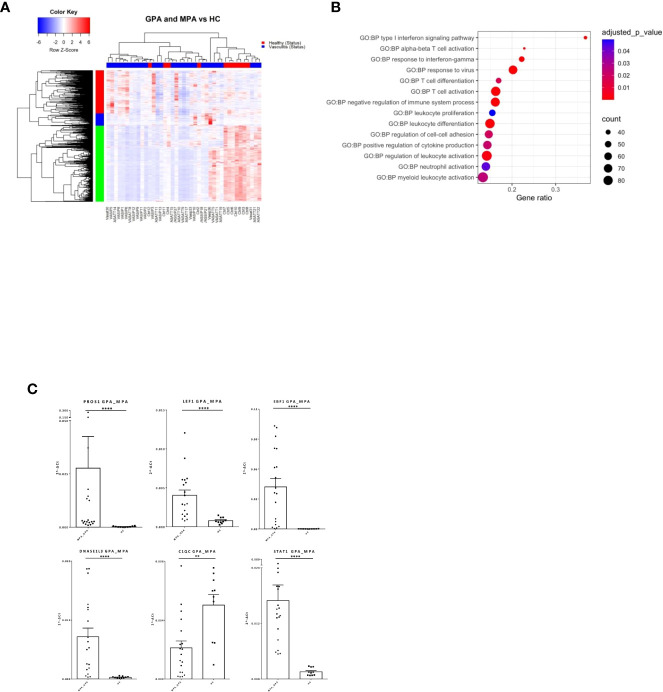
Distinct transcriptional profile and biological processes in AAV patients. Whole blood transcriptional profiling by RNA sequencing of patients with AAV *vs* healthy controls. **(A)** Heatmap of DEGs (p value <0.05) in whole blood of patients with AAV *vs* healthy controls by unsupervised hierarchical clustering. **(B)** Functional enrichment analysis. **(C)** Validation of RNA sequencing with qPCR of selected genes in an independent cohort of AAV patients (Mann Whitney test, two-tailed, p value<0.05). **p ≤ 0.01 and ****p ≤ 0.0001.

AAV comprise of various clinical phenotypes but a detailed molecular map of their common molecular basis is yet to be defined. Comparison of the blood transcriptomes of AAV patients versus healthy controls revealed a “pan-vasculitis” signature comprising 1,982 differentially expressed genes (DEGs) (**Figure 1A**; **Supplementary Table 2**). DEGs related to neutrophil degranulation, type I interferon (IFN) signaling and aberrant T-cell responses were overrepresented in gene ontology analysis (**Figure 1B**). To validate our findings, qPCR was performed in an independent cohort of AAV and healthy individuals. Transcription factors such as STAT1, EBF1, LEF1 and immune/complement related genes such as PROS1 and C1QC are among the validated genes (**Figure 1C**).

### GPA is characterized by aberrant type I interferon and neutrophil degranulation gene signatures

**Figure 2:**
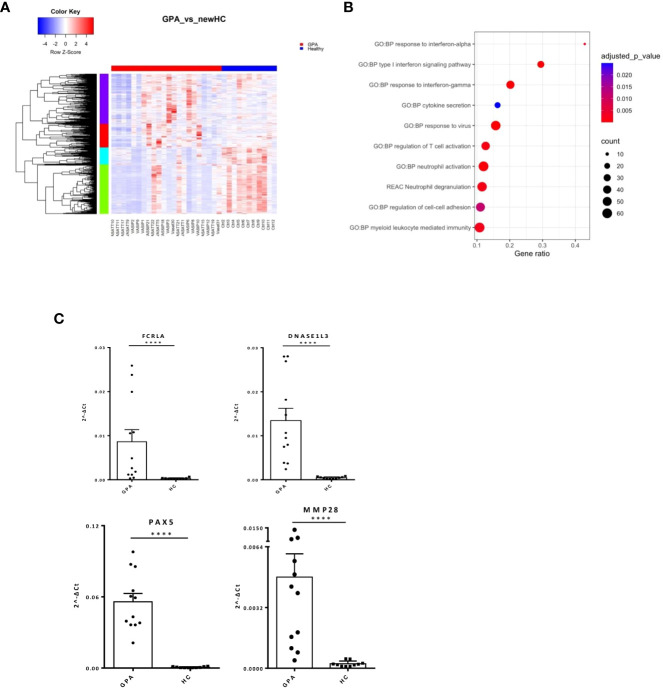
Neutrophil degranulation and type I IFN signaling characterize the GPA transcriptional map. Whole blood transcriptional profiling by RNA sequencing of patients with GPA *vs* healthy controls. **(A)** Heatmap of DEGs (p value <0.05) in whole blood of patients with GPA and healthy controls by unsupervised hierarchical clustering. **(B)** Functional enrichment analysis of the deregulated pathways in GPA. **(C)** Validation of RNA sequencing with qPCR of selected genes in an independent cohort of GPA patients(Mann Whitney test, two-tailed, p value<0.05). ****p ≤ 0.0001.

To define GPA-specific gene signatures, the transcriptome of GPA patients was compared with that of matched healthy individuals yielding 1,319 DEGs (**Figure 2A**; **Supplementary Table 3**). Pathways involved in type I IFN and IFN-γ signaling as well as neutrophil mediated immune responses were extensively deregulated in GPA (**Figure 2B**). We identified IRF8, IRF1, STAT3, GATA1, GATA2 as putative upstream regulators of the GPA signature (**Supplementary Figure 1A**).

Unsupervised hierarchical clustering revealed four patterns of expression among DEGs. In addition to the aforementioned perturbations, enrichment analysis of a 222-gene cluster underscored IL1b mediated responses in GPA pathogenesis (**Supplementary Table 4**). Interestingly, gene network representation of the 222-gene cluster identified genes related to IFN signaling, such as STAT1, ISG15, IFIT3, IFITM1, TRIM22 and histocompatibility genes, such as HLA-E, HLA-F as hub genes (**Supplementary Figure 1B**). qPCR of key genes in the independent patient and healthy cohorts resulted in validation of immune-related (FCRLA, MMP28, DNASE1L3) and developmental genes (PAX5) (**Figure 2C**).

These data suggest that broad type 1 IFN, IFN-γ and innate immunity deregulations may contribute to GPA initiation and progression.

### MPA is characterized by transcriptome aberrations related to neutrophil degranulation, autophagy and mRNA splicing

**Figure 3:**
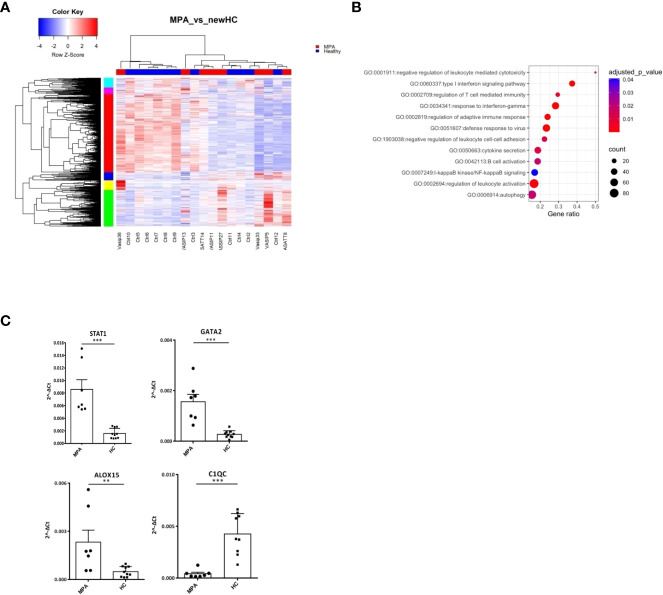
MPA transcriptional networks implicate autophagy and mRNA splicing. Whole blood transcriptional profiling by RNA sequencing of patients with MPA *vs* healthy controls. **(A)** Heatmap of DEGs (p value <0.05) of patients with MPA and healthy controls by unsupervised hierarchical clustering. **(B)** Functional enrichment analysis of the deregulated pathways in MPA. **(C)** Validation of RNA sequencing with qPCR of selected genes in an independent cohort of MPA patients (Mann Whitney test, two-tailed, p value<0.05). **p ≤ 0.01 and ***p ≤ 0.001.

Next, we characterized the transcriptome of MPA patients. A total of 2,326 DEGs were detected (**Figure 3A**; **Supplementary Table 5**), enriched in B and T cell mediated immunity, type I IFN and IFNγ, NF-kB signaling and autophagy (**Figure 3B**). Transcription factors involved in ribosomal RNA (rRNA) transcription, transcription initiation and epigenetic modifications, such as UBTF, TAF1 (40), YY1 (41) or in IFN response, such as IRF8, STAT3 (42) were identified as potential upstream regulators (**Supplementary Figure 2A**). Kinases predicted to regulate DEGs were also defined (**Supplementary Table 6**); the druggable kinase targets, glycogen synthase kinase 3 beta (GSK3B) and casein kinase II subunit alpha (CSNK2A1) were predicted to act as regulators of 53 and 49 substrates of the DEGs input, respectively.

To further elucidate the mechanisms that dictate MPA, gene clusters of pathogenetic importance were identified. Perturbations of mRNA splicing were uncovered by the enrichment analysis of a 101-gene cluster (**Supplementary Figure 2B**). Additionally, a “neutrophilic” cluster of 572 genes, including DNASE1L1, ALOX5, PTAFR, CXCR1, CPPED1, FCGR2B (**Supplementary Figure 2C**) and a cluster of mainly IFN related genes, such as IFI35, OAS1, STAT2, MX2, USP18, IRF7, IFIT1, IFITM1, STAT1, IRF1 were found (**Supplementary Figure 2D**). We confirmed by qPCR several transcription factors (STAT1, GATA2), metabolic (ALOX15) and complement related genes (C1QC) (**Figure 3C**).

These data suggest that type I IFN signaling, neutrophil degranulation and mRNA processing pathways represent three robust signals in MPA.

### Deregulations of cell cycle checkpoints, neutrophil degranulation and oxidative phosphorylation define transcriptional landscape of active AAV

**Figure 4:**
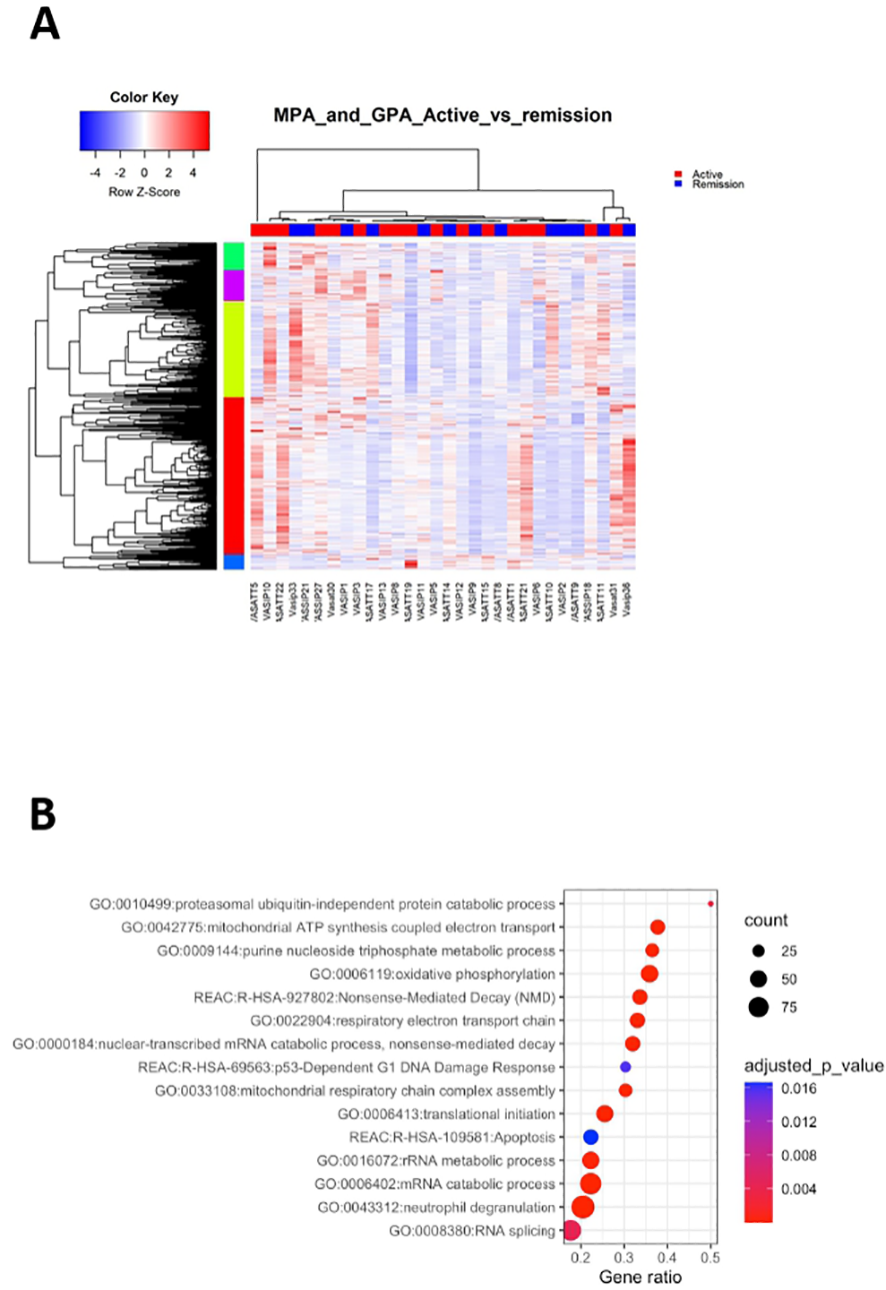
Active disease is accompanied by cell cycle and metabolic regulation. Whole blood transcriptional profiling by RNA sequencing of patients with active AAV *vs* AAV in remission. **(A)** Heatmap of DEGs (p value <0.05) of patients with active AAV and AAV in remission by unsupervised hierarchical clustering. **(B)** Functional enrichment analysis of the deregulated pathways in active AAV cohort.

By comparing the transcriptional profile of active AAV patients versus their counterparts in remission, we identified 2,373 DEGs (**Figure 4A**; **Supplementary Table 7**).

Pathways related to neutrophil activation, TNF-mediated signaling, antigen processing and presentation were overrepresented in active disease (**Figure 4B**; **Supplementary Table 8**). Furthermore, genes involved in IFN signaling, including IFI35, IFI27, ISG20, HLA-F, HLA-A, HLA-B showed higher expression in active disease. Biological processes linked to cell cycle checkpoints regulation, such as p53-independent DNA damage response were enriched in active status. Of note genes encoding proteasome components, such as PSMA5, PSMD13, PSMC5, PSMB6, PSMB1 and ubiquitin (UBB), were upregulated in active disease, suggesting that DNA damage-induced alterations of proteasome proteolytic activity might be implicated with active disease (43). Finally, aberrancies of cellular biochemistry and metabolism (**Figure 4B**) or mRNA surveillance mechanisms, such as nonsense-mediated decay (NMD) were reflected in the active disease transcriptome.

To cope with the complexity of the gene expression data, the X2K computational pipeline was used (44). Several pleiotropic transcription factors including ELF1, CREB1, FLI1, MYC, NFYB, PML were detected (**Supplementary Table 9**). Interestingly, impaired expression of FLI1 has been implicated with other autoimmune disorders, such as SLE (45), whereas the CREB transcription factor family plays a role in the development and maintenance of Tregs (46). KEA returned 134 enriched kinases, including mitogen-activated protein kinase 1 (MAPK1), cyclin dependent kinase 4 (CDK4), homeodomain interacting protein kinase 2 (HIPK2) and Janus kinase 2 (JAK2) (**Supplementary Table 10**).

Together, deregulation of processes related to neutrophil degranulation, cell cycle progression and metabolism efficiently differentiated active disease, suggesting that restoration of their function might be linked with remission induction.

### Humoral immunity gene expression signatures correlate with ANCA positivity

**Figure 5:**
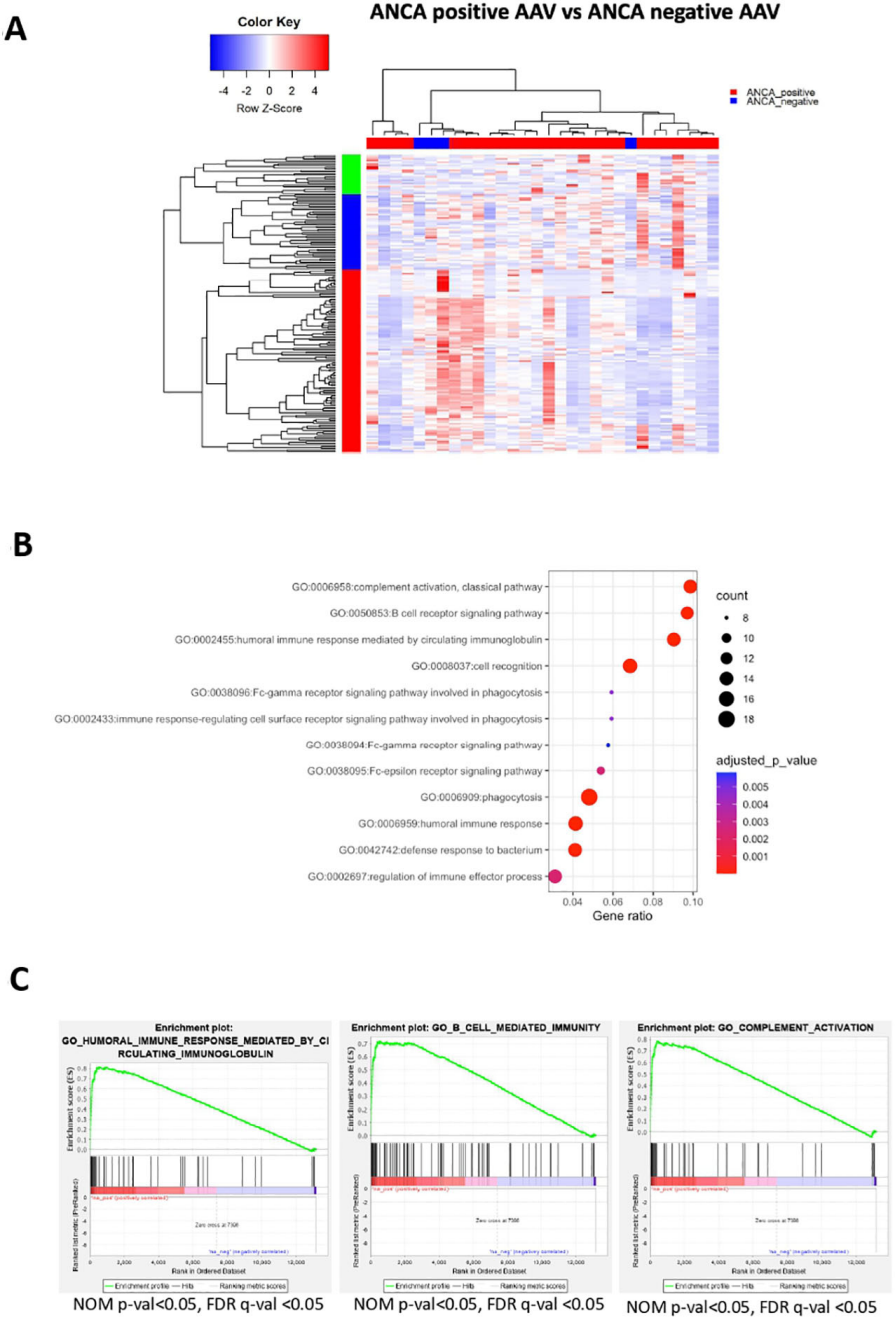
ANCA positivity is characterized by upregulation of genes related to humoral immunity. Whole blood transcriptional profiling by RNA sequencing of AAV patients according to their ANCA status **(A)** Heatmap of DEGs (p value <0.05) of ANCA positive *vs* ANCA negative patients by unsupervised hierarchical clustering. **(B)** Functional enrichment analysis of the upregulated pathways in ANCA positive patients. **(C)** Ranked GSEA and leading-edge analysis to determine genes with high impact on humoral immune responses.

ANCA are implicated in AAV pathogenesis and furthermore, ANCA-positive patients display a different clinical course and response to therapies compared to ANCA-negative patients. Whether ANCA positivity is accompanied by specific transcriptional signature remains elusive. By comparing the blood transcriptome of ANCA-positive with ANCA-negative patients, 182 DEGs were identified (**Figure 5A**; **Supplementary Table 11**). Pathways related to phagocytosis, activation of classical complement pathway, Fc-gamma receptor signaling and BCR activation were prominent in upregulated DEGs (**Figure 5B**). To determine genes with high impact on humoral immune responses, ranked GSEA and leading-edge analysis were performed (**Figure 5C**). Genes encoding constant and variable domains of immunoglobulin heavy chains, such as IGHE, IGHV3-23, IGLV7-43, IGLV7-43 contributed largely to the core enrichment.

To prioritize the upregulated DEGs, a GeneMANIA-based weighted interaction network was created (**Supplementary Figure 3A**). Highly interconnected nodes included, among others, genes essential for cytoskeleton organization and cell motility, such as ACTN4, MYH9, FLNA and TRRAP (47, 48). To capture a more detailed picture of the gene expression regulation, a X2K network corresponding to upregulated DEGs was constructed (**Supplementary Figure 3B**). Briefly, transcription factors, including GATA1, GATA2, SMAD4, NFE2L2, FOS were predicted to orchestrate gene activity.

GWAS analysis of patients with AAV shows that its pathogenesis has genetic component, distinguishing GPA from MPA as well as implying that PR3-AAV and MPO-AAV are distinct autoimmune syndromes, independent of the characteristics of the clinical phenotype (11). Therefore, this genetic impact on the phenotypes of AAV patients would probably be reflected on the respective transcriptomes. To address this question, we performed differential gene expression analysis based on the antibody specificity of the patients. MPO-ANCA positive transcriptomes (n=15) were compared to PR3-ANCA positive transcriptomes (n=11). Ultimately, 155 genes (104 upregulated) were differentially expressed between these two groups of patients (**Supplementary Figure 4A**, **Supplemental Table 12**). GSEA of the DEGs that dissect MPO+/PR3+ AAV patients pinpoints that they participate in complement activation, humoral immune response through circulating immunoglobulins, response to type I IFN, metabolism through oxidative phosphorylation and production of ROS (**Supplementary Figure 4B**; **Supplemental Table 13**).

Finally, we conclude that transcriptional signatures linked to aberrant humoral responses constitute a distinct characteristic of ANCA positive AAV. ANCA(+)*vs*(-) is discriminating molecular phenotype of the patients much more robustly than MPO(+)*vs*PR3(+).

### Co-expression analysis revealed four AAV molecular endotypes with distinct gene expression signatures

**Figure 6:**
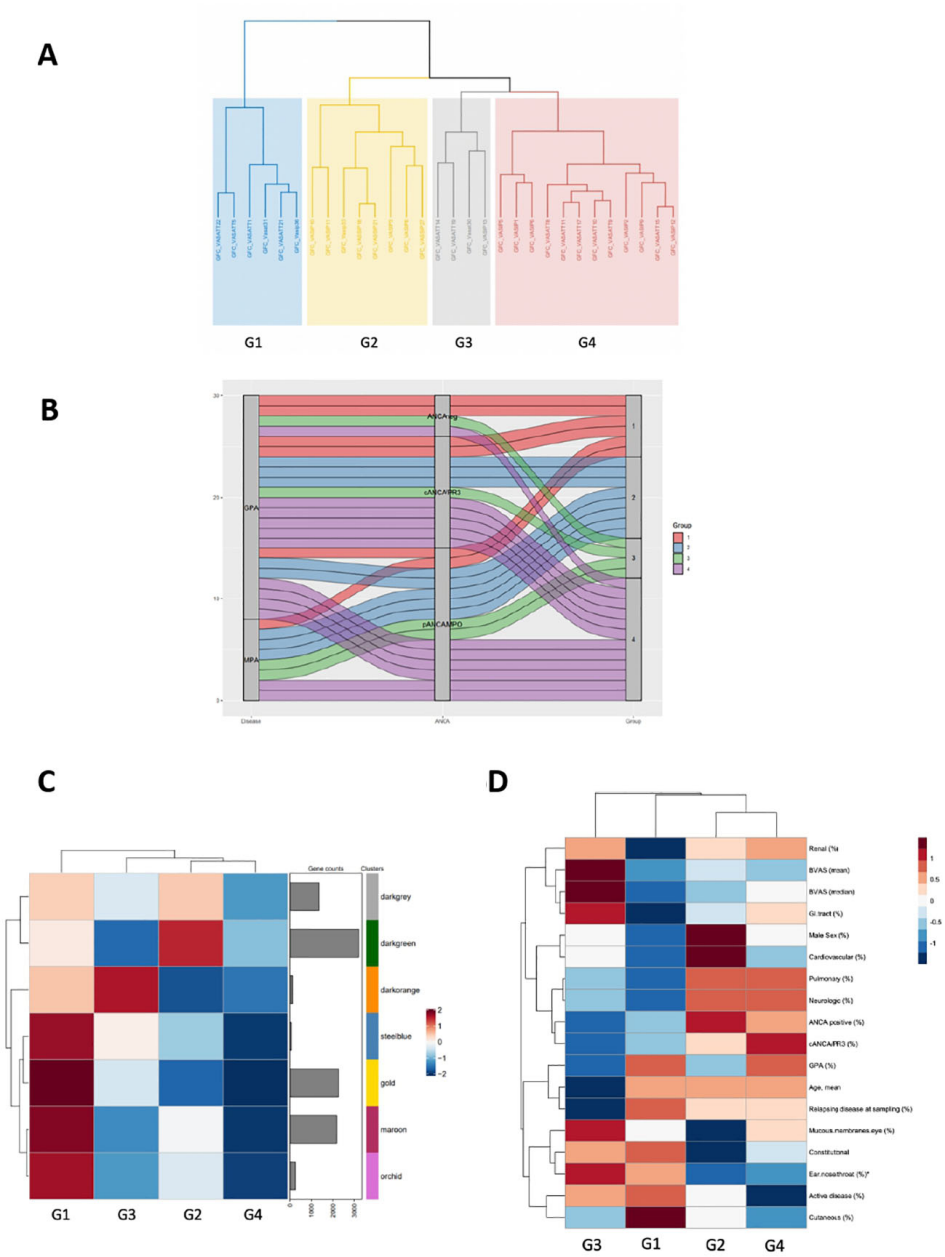
Co-expression analysis of the AAV transcriptome defines distinct transcriptional modular patient clusters. **(A)** Hierarchical clustering of the samples according to their group fold changes (GFCs) for each module, generated four groups of samples (G1-G4). **(B)** Alluvial diagram showing the regrouping of patients according to co-expressed transcripts. **(C)** Heatmap demonstrating the mean of the GFCs of the gene modules – identified by the CoCena2 analysis - in each one of the defined patient groups. Enhanced expression of the orchid, maroon, gold and steelblue modules distinguished G1. Enrichment of the darkorange module characterized G3. Increased expression of the darkgreen module was indicative of G2. **(D)** Heatmap depicting the prevalence of the each AAV subtype, the distribution of the clinical and demographic features and the frequency of active disease across patient groups. *:p<0.05 in Kruskal-Wallis test, Chi-squared test.

Conventional differential expression analysis based on clinical classification often fails to fully explain molecular heterogeneity underlying immune responses in AAV. By contrast, co-expression analysis can facilitate a data-driven, clinically independent regrouping of samples. Using the CoCena² pipeline in our AAV dataset, seven co-expression modules, which were represented by color dark grey to orchid were defined (**Supplementary Figure 5A**). Hierarchical clustering of the samples according to their group fold changes (GFCs) for each module, generated four groups of samples (G1-G4) (**Figures 6A**, **B**). To investigate the molecular basis of the applied re-stratification strategy, the enrichment of each newly defined group was examined (**Figure 6C**). Higher expression of orchid module, which contained genes involved in neutrophil degranulation, B cell mediated responses and complement activation distinguished G1 (**Supplementary Figure 5B**). G3, encompassing mainly patients with high BVAS score and upper respiratory tract involvement (**Figure 6D**), was characterized by enrichment of the 131-gene erythropoiesis and platelet degranulation related dark orange module, along with dampening of the neutrophilic signature expression. Strikingly, genes of integrin family (ITGB3, ITGA2B, ITGB5), essential for neutrophil recruitment into inflamed tissues and phagocytosis were present in the dark orange module. The ANCA positive group G2, which was clinically discriminated by increased prevalence of pulmonary involvement, showed heightened expression of the dark green module. Detailed functionally enrichment analysis of the dark green module disclosed extensive deregulation of processes associated, among others, with oxidative phosphorylation and neutrophil activation.

Collectively, co-expression analysis suggested the presence of distinct AAV molecular endotypes.

### Neutrophil and IFN-related pathways differences between SLE and AAV

Despite its aggressive course, in AAV responses to existing therapies are more solid than those in SLE. Comparison of the transcriptomic landscape of both diseases may provide an unbiased, comprehensive look of the underlying pathogenetic mechanisms and explain the differences in the natural course and response to treatment.

**Figure 7:**
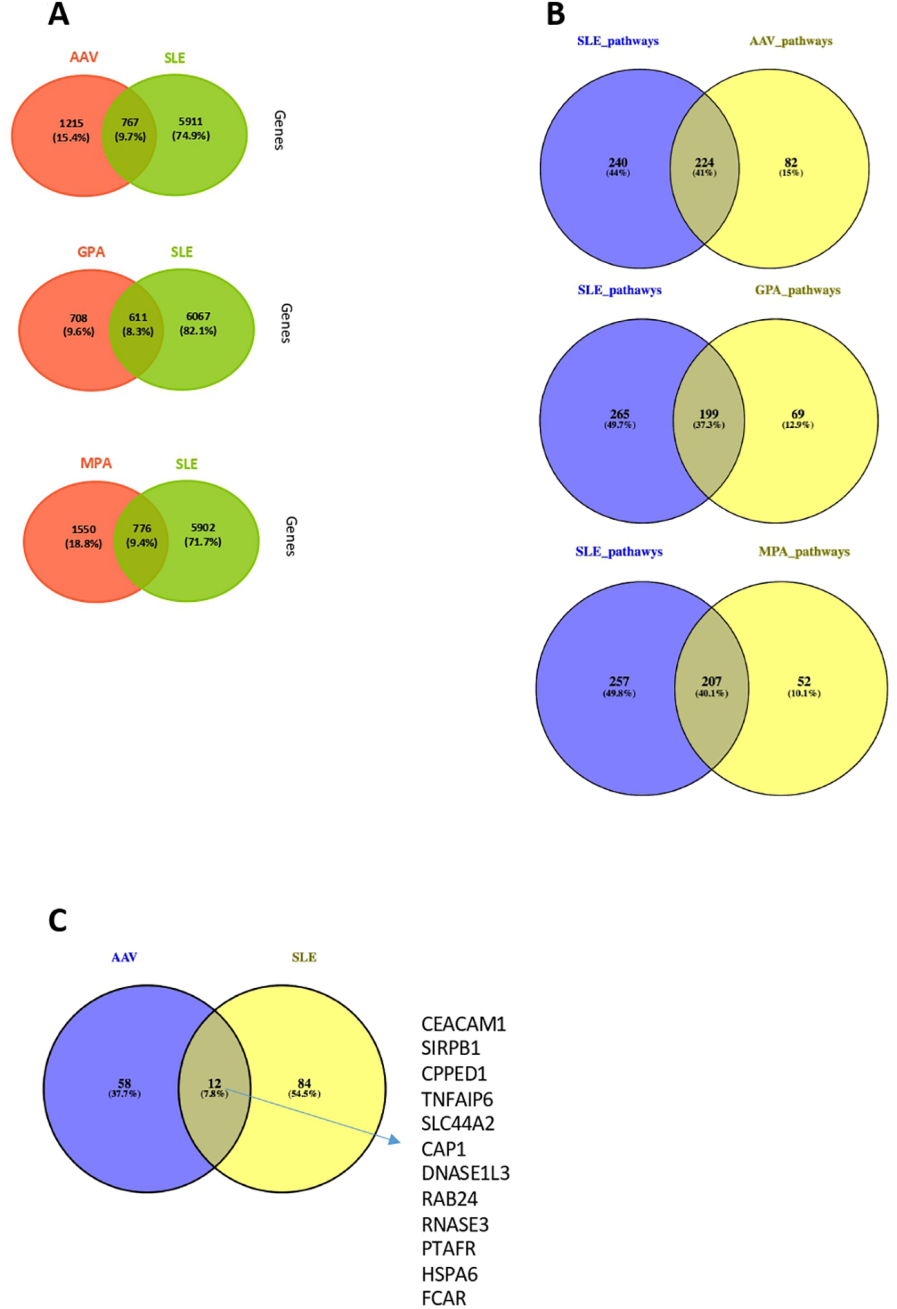
Comparison of whole blood transcriptome and neutrophil signature between AAV and SLE patients. **(A)** Venn diagrams representing the overlap between DEGs in AAV *vs* SLE, GPA *vs* SLE and MPA *vs* SLE. **(B)** Venn diagrams representing the overlap between involved pathways derived from gene ontology in AAV *vs* SLE, GPA *vs* SLE and MPA *vs* SLE. **(C)** Comparison at gene level between SLE and AAV neutrophilic signatures of these two gene sets.

We investigated whether an overlap - in terms of DEGs and pathogenetic pathways – is present between SLE (20) and AAV (GPA or MPA). The comparison showed that 41%, 37% and 40% of the pathways derived of SLE DEGs, were also detected in the “Pan-vasculitis”, MPA and GPA gene expression signatures, respectively. (**Figures 7A**, **B**). GPA and SLE shared 199 enriched pathways (type I and II IFN signaling, neutrophil degranulation, cytokine signaling), whereas 207 biological processes were impaired both in SLE and MPA (metabolic pathways, autophagy, RNA metabolism and processing) (**Figure 7B**).

Since deregulations of neutrophils have emerged as a crucial driver of SLE and AAV pathogenesis (17, 49–52), we examined whether the neutrophil activation associated signatures qualitatively differ between the two clinical entities. To this end, the DEGs belonging to the SLE neutrophilic signature were compared with the genes defining the AAV neutrophil signature. Of note, only a weak transcriptional overlap was observed between the SLE and AAV neutrophilic signatures, suggesting that distinct mechanisms underly neutrophilic inflammation in SLE and AAV (**Figure 7C**).

## Discussion

Despite advances in the understanding of the molecular mechanisms underlying AAV, the disease etiopathogenesis remains elusive. Herein, we identified aberrant IFN and neutrophil transcriptional responses associated with GPA and MPA. Deregulation of cell cycle checkpoints control, aberrancies of neutrophil function and cellular metabolism defined a status of active disease, while inappropriate humoral responses were related to ANCA positivity. By high-throughput computational methods, we re-stratified AAV patients based on their gene signatures, regardless of their clinical annotation. Finally, leveraging one of the largest SLE RNA sequencing cohort to date, we systematically explored the transcriptional similarities and differences between SLE and AAV, reporting more homogeneity and less “disorganization” in the AAV transcriptome.

Neutrophil activation and degranulation play a key role in AAV pathogenesis. ANCA-activated neutrophils generate ROS, release destructive enzymes and extrude NETs at the site of inflammation. Accordingly, augmented expression of the granulocyte gene signature is related to active disease and insufficient response to treatment (17, 49–52). Patients with active AAV displayed a robust transcriptomic neutrophilic signature. Low-density granulocytes (LDGs) – a distinct subset of neutrophils – exhibit increased capacity to form NETs (23, 53), suggesting that this cell type is likely to serve as a major source of the identified neutrophilic signature.

Necrotizing granuloma formation is a distinct feature of GPA. Interestingly, PR3-maturated dendritic cells from GPA patients prime robust Th1 responses of PR3-specific CD4+ T cells, which in turn produce large amounts of IFNγ (54). It is tempting to speculate that enrichment of IFNγ related pathways in GPA blood transcriptome might account -at least in part- for the effect of PR3 on the functional maturation of dendritic cells.

Immunometabolism has emerged as a central mechanism for the regulation of adaptive and innate immune responses. In our study, patients with active disease exhibited disturbances of pathways involved in mitochondrial respiratory chain. Although neutrophils display limited reliance on oxidative phosphorylation at baseline, there is compelling evidence for the role of oxidative phosphorylation in NETosis and chemotaxis (55). The latter, coupled with the fact that excessive NET formation is present in active disease (17, 56) provides a reasonable interpretation of our findings.

Chronic inflammation favors genomic instability and DNA damage, setting DNA damage response and repair (DDR/R) in motion (57–60). Oxidative stress, characterized by excessive production and defective removal of ROS is a well-defined cause of DNA damage, leading to single-strand breaks, double-strand breaks and oxidized purines and pyrimidines (61). We are proposing that enrichment of cell cycle related pathways found in patients with active disease, might reflect a cellular response to DNA lesions, elicited by increased release of ROS by hyperactivated neutrophils (17, 49–52). Interestingly, genes related to type I IFN signature, including IRF3 were upregulated in active AAV, suggesting that accumulation of DNA lesions followed by induction of the cGAS-STING (stimulator of IFN genes)-IRF3 pathway and production of type I IFN (62) might be operant.

Clinical classification of AAV often fails to comprehensively recapitulate the mechanistic heterogeneity of the disease. Using co-expression network analysis, we re-grouped the AAV patients in an unbiased, data-driven manner. Neutrophil activation transcriptional signature defined G1, corroborating the molecular taxonomy findings of Gill et al. (24) Pathways reflecting neutrophil activation were not uniformly upregulated across the several endotypes, suggesting that additional mechanistic drivers might be present in AAV. Dysregulation of the mitochondrial function/oxidative phosphorylation dominated in patient group G2, whereas G3 demonstrated a transcriptional pattern indicative of platelet activation and erythropoiesis. Together, our data suggest that the transcriptome defined endotypes are not apparent with the current clinical classification or serologic status.

Defining gene expression signatures that differentiate AAV from SLE is fundamental for the development of accurate diagnostic biomarkers and might explain the differences in response to therapy and risk of flares between these diseases. By applying an unsupervised, molecular taxonomy approach (20, 63), we have previously highlighted the broad heterogeneity, extensive fragmentation and wide reorganization of transcription in SLE (64). In contrast, the data-driven re-stratification of AAV demonstrated a significantly less extensive fragmentation of the AAV dataset. Although differences in sample size may affect our findings, a relative homogeneity of disease driving mechanisms in AAV, likely to favorably influence rates of response to treatment could be implied.

SLE and AAV are both characterized by a strong neutrophilic transcriptional signature. Interestingly, this signature exhibited important quantitative and qualitative differences between these diseases, suggesting that distinct pathophysiological mechanisms might orchestrate neutrophilic inflammation in SLE and AAV at different stages of the disease.

Our study has certain limitations. The vast majority of patients were receiving immunosuppressive treatment at sampling, including corticosteroids. Limitation associated with whole blood transcriptomic analysis, such as cellular heterogeneity should also be taken into consideration. As this study included only Caucasians, generalization of our results to other ethnic groups is questionable. We also recognize that the number of involved patients could be larger. However, our total cohort of AAV patients is similar to other prominent studies in the field. Second, the number of recruited patients is dependent on the AAV prevalence and, even though AAV are rare diseases, approximately one quarter of the AAV patients recruited in our multicenter registry were included in the current study. Third, the analytical power of RNA-sequencing can map both quantitative and qualitative dimensions of gene expression in an absolute statistically significant manner even with relatively limited number of patients. Of interest, while almost three out of four patients of our cohort had GPA, the respective prevalence of PR3, MPO and negative ANCA in this subset of patients was 50%, 32% and 18%. The subset of AAV patients with MPO-positive GPA is not uncommon and it has been reported in 16% of patients, while other observational studies have reported even higher prevalence (65–68). Given the relatively small number of included patients, we cannot rule out the possibility of a selection bias. Another limitation of this study is summed in the fact that no serial sampling were included so as to assess the disease trajectory as to activity and response to therapy. Finally, analysis of the transcriptome at the single cell level has added great amount of information about the pathogenic molecular landscape of various diseases, including autoimmune ones (69, 70). Single-cell studies in different vasculitides have already shed light on the pathogenesis of each entity, but for the time being they have focused on Takayasu arteritis (71), Behcet’s Disease (72) and Kawasaki Disease (73, 74). Heterogeneity is analyzed in a certain degree in this study, as RNA-sequencing technology utilized is bulk.

In summary, our data provide a comprehensive assessment of the transcriptomic landscape of the human AAV in an unbiased way without preconceived notions, providing novel evidence for its key differences from the SLE transcriptome. In this context, we provide additional insights into the pathogenesis, monitoring and potential targets of therapy.

## Data availability statement

Original datasets are available in a publicly accessible repository: The original contributions presented in the study are publicly available. The data presented in the study are deposited in the EGA repository, accession number EGAS00001006704, under the link https://ega-archive.org/studies/EGAS00001006704.

## Ethics statement

The study was approved by the institutional review boards of 2 hospitals (HGH: 57/26-03-2018/AUH: EDB 103/06-03-2014). The patients/participants provided their written informed consent to participate in this study.

## Author contributions

AB, KT and PG designed and performed the experiments, analysed data, generated figures and wrote the manuscript. AF, NM and DN analysed data and generated figures. NM performed experiments. KT, DP, AP, AC, AGP and PG performed clinical evaluation of patients, provided human specimens and interpreted data. GB participated in the design, the interpretation of data and the editing of the manuscript. DTB and DV supervised the study and the writing of the manuscript. AB, KT and PG contributed equally. DB and DV contributed equally. All authors contributed to the article and approved the submitted version.

## Supplementary material

Supplementary Figure 1
**(A**) Transcription factors predictive of regulating the GPA gene expression profile according to the ChIP Enrichment Analysis (ChEA). IRF1, STAT3 and GATA1 were identified as potential upstream regulators. **(B**)Gene network representation of a 222-gene cluster derived from the comparison of GPA with healthy individuals. Transcripts related to IFN signaling as well as histocompatibility genes emerged as hub genes.

Supplementary Figure 2
**| (A)** Putative upstream regulators of the MPA signature according to the ChIP Enrichment Analysis (ChEA). Transcription factors associated with ribosomal RNA (rRNA) transcription, transcription initiation and epigenetic modifications were identified as essential regulators of the MPA transcriptional profile. **(B**) Enrichment map of the biological processes resulted from the functional enrichment analysis of a 101-gene cluster derived from the comparison of MPA with healthy individuals. Terms related to mRNA splicing were found – among others - to be significantly enriched. **(C**) Enrichment map of the biological processes resulted from the functional enrichment analysis of a 572-gene cluster derived from the comparison of MPA with healthy individuals. Pathways associated with neutrophil degranulation dominated in this gene-cluster. **(D**) Enrichment map of the biological processes resulted from the functional enrichment analysis of a gene cluster derived from the comparison of MPA with healthy individuals. Genes related to IFN responses were overrepresented among the DEGs of this gene cluster.

Supplementary Figure 3 (**A)** Gene network representation of the upregulated DEGs resulted from the comparison of ANCA positive patients with ANCA negative patients. Genes related to cytoskeleton organization and cell motility emerged as hub genes.**(B**) X2K Web based gene interaction network of the upregulated DEGs resulted from the comparison of ANCA positive patients with ANCA negative patients, inferred using the findings from transcription factor enrichment analysis, protein-protein interaction network analysis, and kinase enrichment analysis. Transcription factors, including GATA1, GATA2, SMAD4, NFE2L2, FOS were identified as potential regulators of the upregulated DEGs.

Supplementary Figure 4 (**A)** Heatmap of DEGs (p value <0.05) of anti-MPO+ve *vs* anti-PR3+ve patients by unsupervised hierarchical clustering.**(B)** Ranked GSEA resulted from the comparison of the whole blood transcriptome of anti-MPO+ve versus anti-PR3+ve patients.

Supplementary Figure 5
**(A)** CoCena2 analysis-based modules (darkgrey to orchid) of commonly regulated transcripts and heatmap demonstrating the group fold changes (GFC) of each sample per module. GFCs of each sample per module were calculated as previously described in Garantziotis et al. [58]. **(B)** Functional enrichment analysis of the CoCena2 analysis derived modules. Briefly, transcripts included in the orchid module were mainly enriched in processes related to neutrophil degranulation and B cell mediated responses. Enrichment analysis of the darkorgange module revealed pathways associated with erythropoiesis and platelet degranulation. Genes of darkgreen module were enriched in oxidative phosphorylation and neutrophil activation.

Supplementary Table 1 Primer list used for the qPCR validation of the literature-curated genes.

Supplementary Table 2 Differentially expressed genes resulted from the comparison of the whole blood transcriptome of patients with GPA or MPA versus healthy individuals.

Supplementary Table 3 Differentially expressed genes resulted from the comparison of the whole blood transcriptome of patients with GPA versus healthy individuals.

Supplementary Table 4 Functional enrichment analysis of the 222-gene cluster identified by the unsupervised hierarchical clustering of the DEGs derived from the comparison of patients with GPA versus healthy individuals.

Supplementary Table 5 Differentially expressed genes resulted from the comparison of the whole blood transcriptome of patients with MPA versus healthy individuals.

Supplementary Table 6 Kinases predictive of regulating the MPA gene expression signature according to the KEA.

Supplementary Table 7 Differentially expressed genes resulted from the comparison of the whole blood transcriptome of active AAV patients versus AAV patients in remission.

Supplementary Table 8 Functional enrichment analysis of the DEGs defining the active AAV gene expression signature.

Supplementary Table 9 Transcription factors predictive of regulating the active disease gene expression signature according to the X2K Web based transcription factor enrichment analysis.

Supplementary Table 10 Kinases predictive of regulating the active disease gene expression signature according to the KEA.

Supplementary Table 11 Differentially expressed genes resulted from the comparison of the whole blood transcriptome of ANCA-positive versus ANCA-negative patients.

Supplementary Table 12 Differentially expressed genes resulted from the comparison of the whole blood transcriptome of anti-MPO+ve versus anti-PR3+ve patients.

Supplementary Table 13 Ranked GSEA analysis resulted from the comparison of the whole blood transcriptome of anti-MPO+ve versus anti-PR3+ve patients.
