# Supplementary figures and images for: The genomic landscape of ANCA-associated vasculitis: Distinct transcriptional signatures, molecular endotypes and comparison with systemic lupus erythematosus
Source: Front Immunol. 2023 Mar 27;14:1072598. doi: 10.3389/fimmu.2023.1072598 (PMC10083368; doi:10.3389/fimmu.2023.1072598)

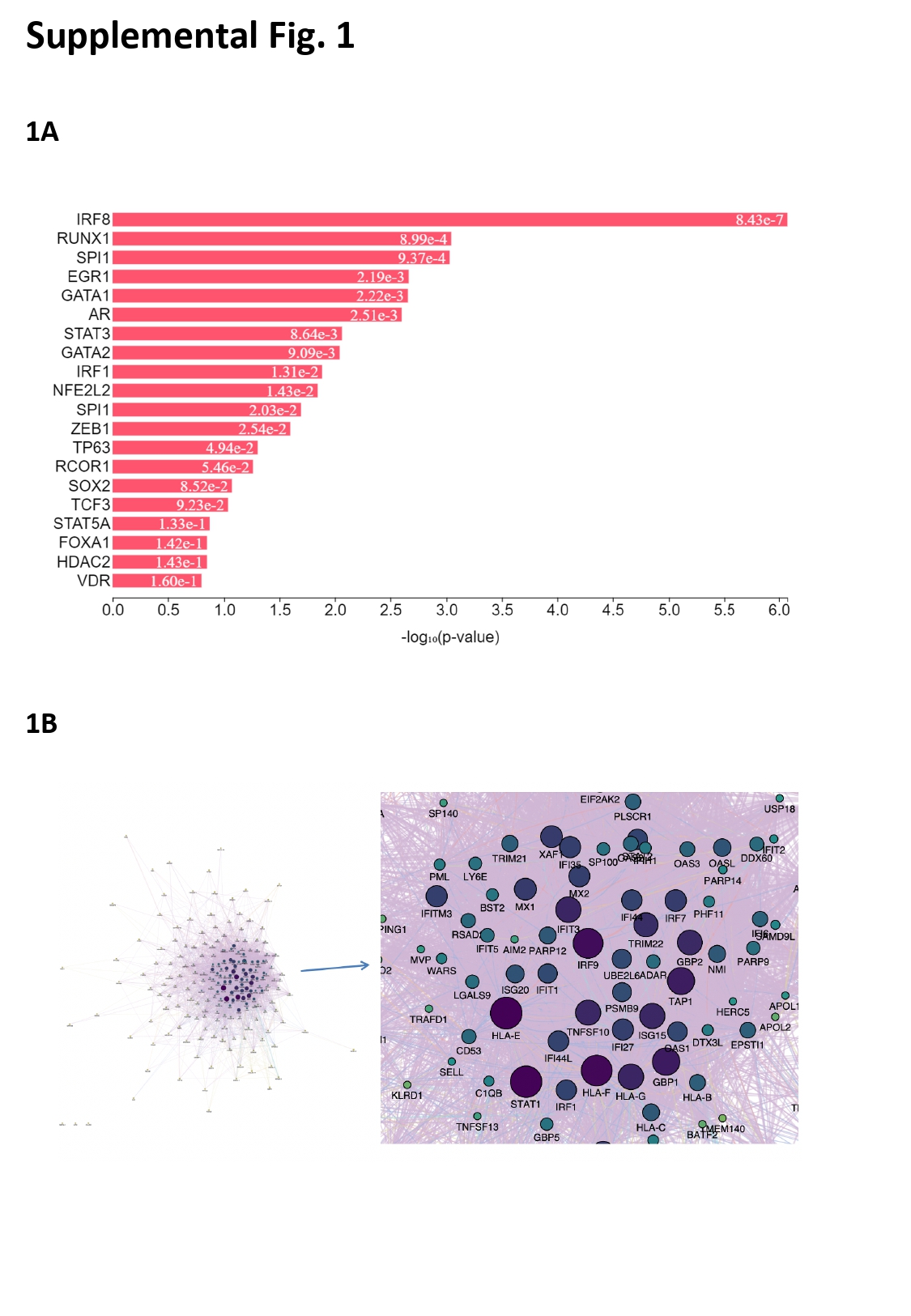

Supplement: Supplementary Figure 1 — (A) Transcription factors predictive of regulating the GPA gene expression profile according to the ChIP Enrichment Analysis (ChEA). IRF1, STAT3 and GATA1 were identified as potential upstream regulators. (B)Gene network representation of a 222-gene cluster derived from the comparison of GPA with healthy individuals. Transcripts related to IFN signaling as well as histocompatibility genes emerged as hub genes. [file Image_1.jpeg]

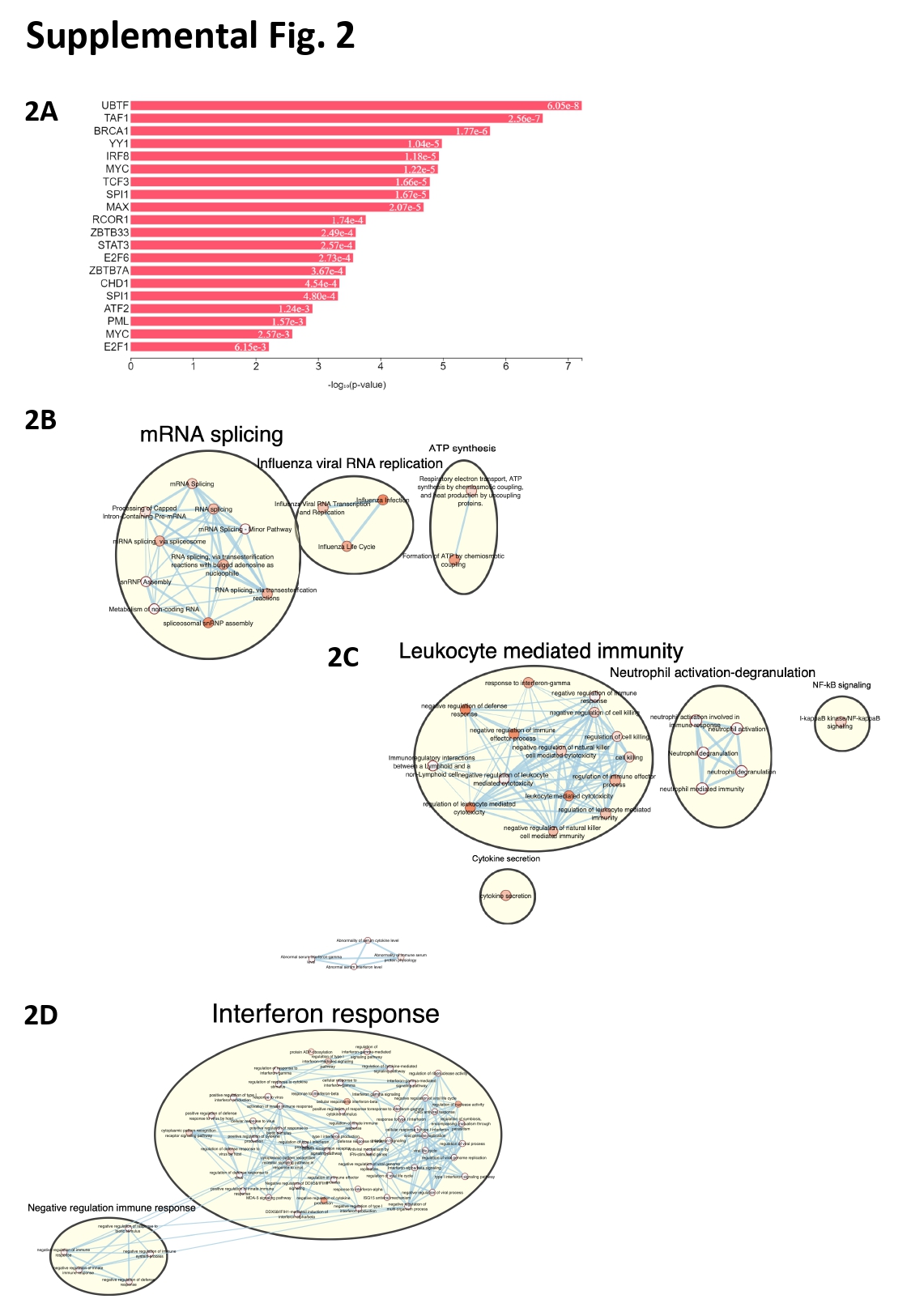

Supplement: Supplementary Figure 2 — | (A) Putative upstream regulators of the MPA signature according to the ChIP Enrichment Analysis (ChEA). Transcription factors associated with ribosomal RNA (rRNA) transcription, transcription initiation and epigenetic modifications were identified as essential regulators of the MPA transcriptional profile. (B) Enrichment map of the biological processes resulted from the functional enrichment analysis of a 101-gene cluster derived from the comparison of MPA with healthy individuals. Terms related to mRNA splicing were found – among others - to be significantly enriched. (C) Enrichment map of the biological processes resulted from the functional enrichment analysis of a 572-gene cluster derived from the comparison of MPA with healthy individuals. Pathways associated with neutrophil degranulation dominated in this gene-cluster. (D) Enrichment map of the biological processes resulted from the functional enrichment analysis of a gene cluster derived from the comparison of MPA with healthy individuals. Genes related to IFN responses were overrepresented among the DEGs of this gene cluster. [file Image_2.jpeg]

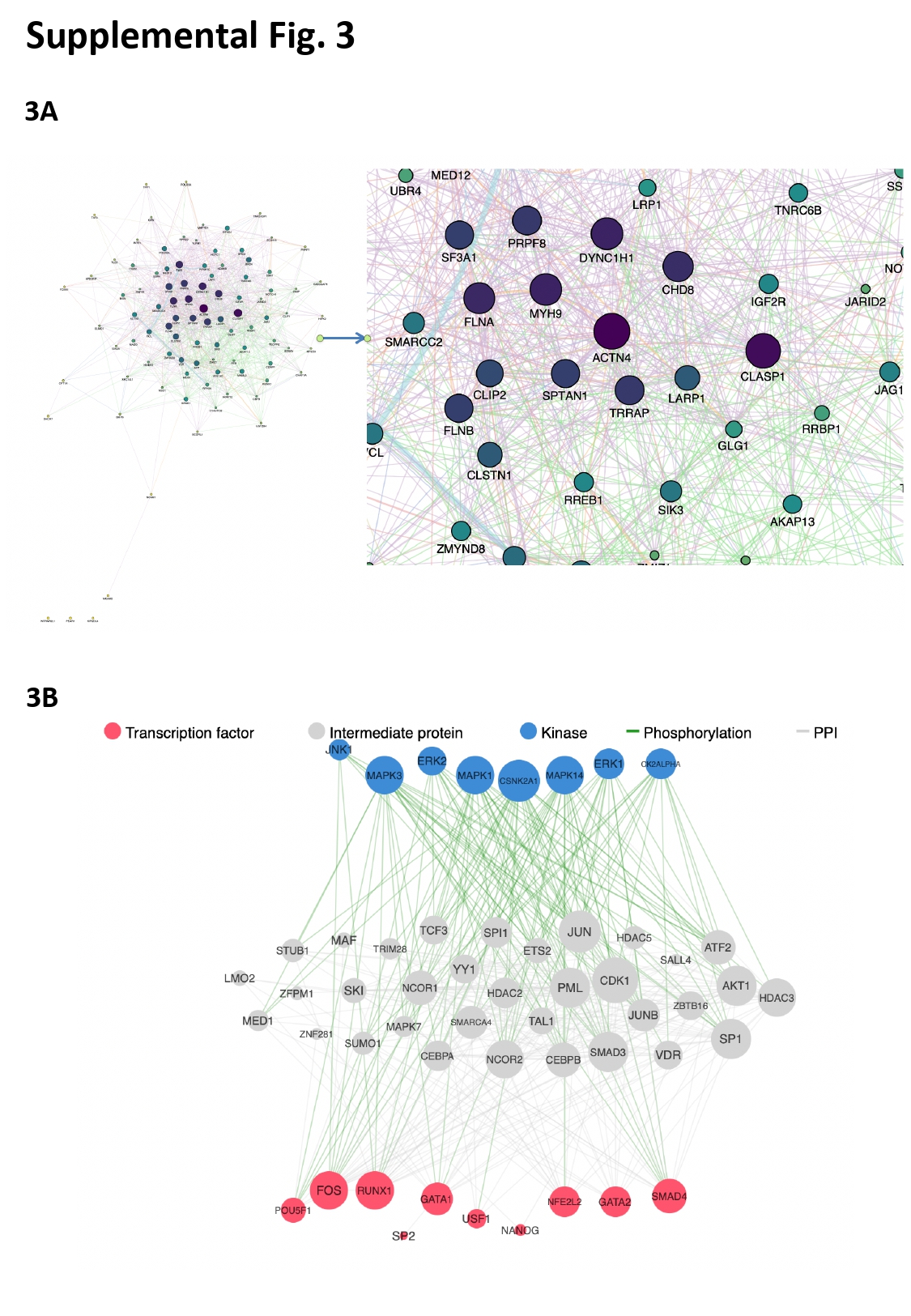

Supplement: Supplementary Figure 3 — (A) Gene network representation of the upregulated DEGs resulted from the comparison of ANCA positive patients with ANCA negative patients. Genes related to cytoskeleton organization and cell motility emerged as hub genes.(B) X2K Web based gene interaction network of the upregulated DEGs resulted from the comparison of ANCA positive patients with ANCA negative patients, inferred using the findings from transcription factor enrichment analysis, protein-protein interaction network analysis, and kinase enrichment analysis. Transcription factors, including GATA1, GATA2, SMAD4, NFE2L2, FOS were identified as potential regulators of the upregulated DEGs. [file Image_3.jpeg]

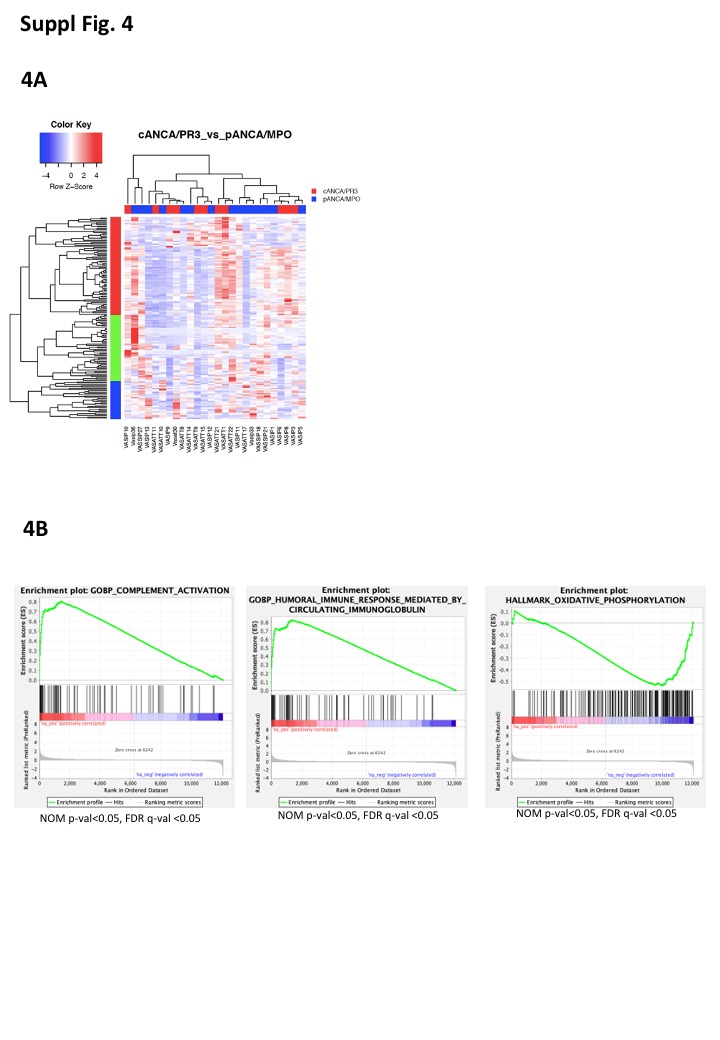

Supplement: Supplementary Figure 4 — (A) Heatmap of DEGs (p value <0.05) of anti-MPO+ve vs anti-PR3+ve patients by unsupervised hierarchical clustering.(B) Ranked GSEA resulted from the comparison of the whole blood transcriptome of anti-MPO+ve versus anti-PR3+ve patients. [file Image_4.jpeg]

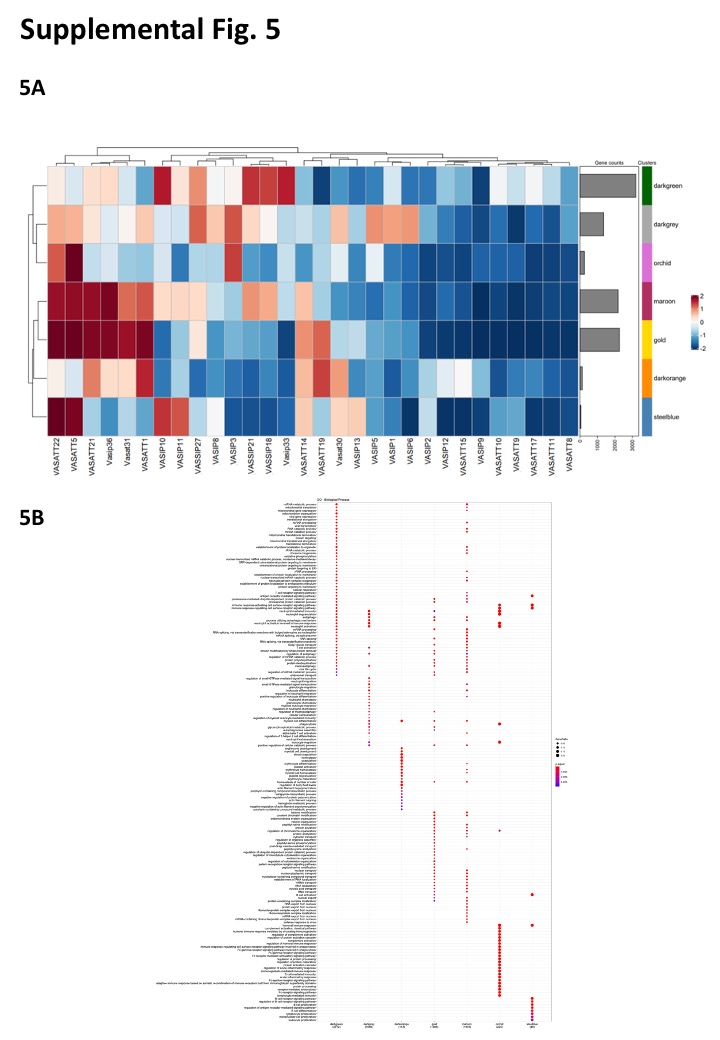

Supplement: Supplementary Figure 5 — (A) CoCena2 analysis-based modules (darkgrey to orchid) of commonly regulated transcripts and heatmap demonstrating the group fold changes (GFC) of each sample per module. GFCs of each sample per module were calculated as previously described in Garantziotis et al. [58]. (B) Functional enrichment analysis of the CoCena2 analysis derived modules. Briefly, transcripts included in the orchid module were mainly enriched in processes related to neutrophil degranulation and B cell mediated responses. Enrichment analysis of the darkorgange module revealed pathways associated with erythropoiesis and platelet degranulation. Genes of darkgreen module were enriched in oxidative phosphorylation and neutrophil activation. [file Image_5.jpeg]
